# Supplementary material for: An allele-selective inter-chromosomal protein bridge supports monogenic antigen expression in the African trypanosome
Source: Nat Commun. 2023 Dec 11;14:8200. doi: 10.1038/s41467-023-44043-y (PMC10713589; doi:10.1038/s41467-023-44043-y)
Supplement: Supplementary file 1 — Supplementary Information [file 41467_2023_44043_MOESM1_ESM.pdf]

Supplementary Materials

An allele-selective inter-chromosomal protein bridge supports monogenic antigen expression in the African trypanosome

Joana R. C. Faria[1,2,3]*, Michele Tinti[1], Catarina A. Marques[1,5], Martin Zoltner[1,6], Harunori Yoshikawa[4,7], Mark C. Field[1,8] & David Horn[1]*

[1]Wellcome Centre for Anti-Infectives Research, Biological Chemistry and Drug Discovery, School of Life Sciences, University of Dundee, UK
[2]Biology Department, University of York, UK
[3]York Biomedical Research Institute, University of York, UK
[4]Gene Regulation and Expression, School of Life Sciences, University of Dundee, UK
[5]Current address: Wellcome Centre for Integrative Parasitology, University of Glasgow, UK
[6]Current address: Faculty of Science, Charles University in Prague, Biocev, Vestec, Czech Republic
[7]Current address: Division of Cell Signaling, Fujii Memorial Institute of Medical Sciences, Institute of Advanced Medical Sciences, Tokushima University, Japan
[8]Biology Centre, Czech Academy of Sciences, Institute of Parasitology, České Budějovice, Czech Republic.

*correspondence and material requests should be addressed to joana.correiafaria@york.ac.uk and d.horn@dundee.ac.uk

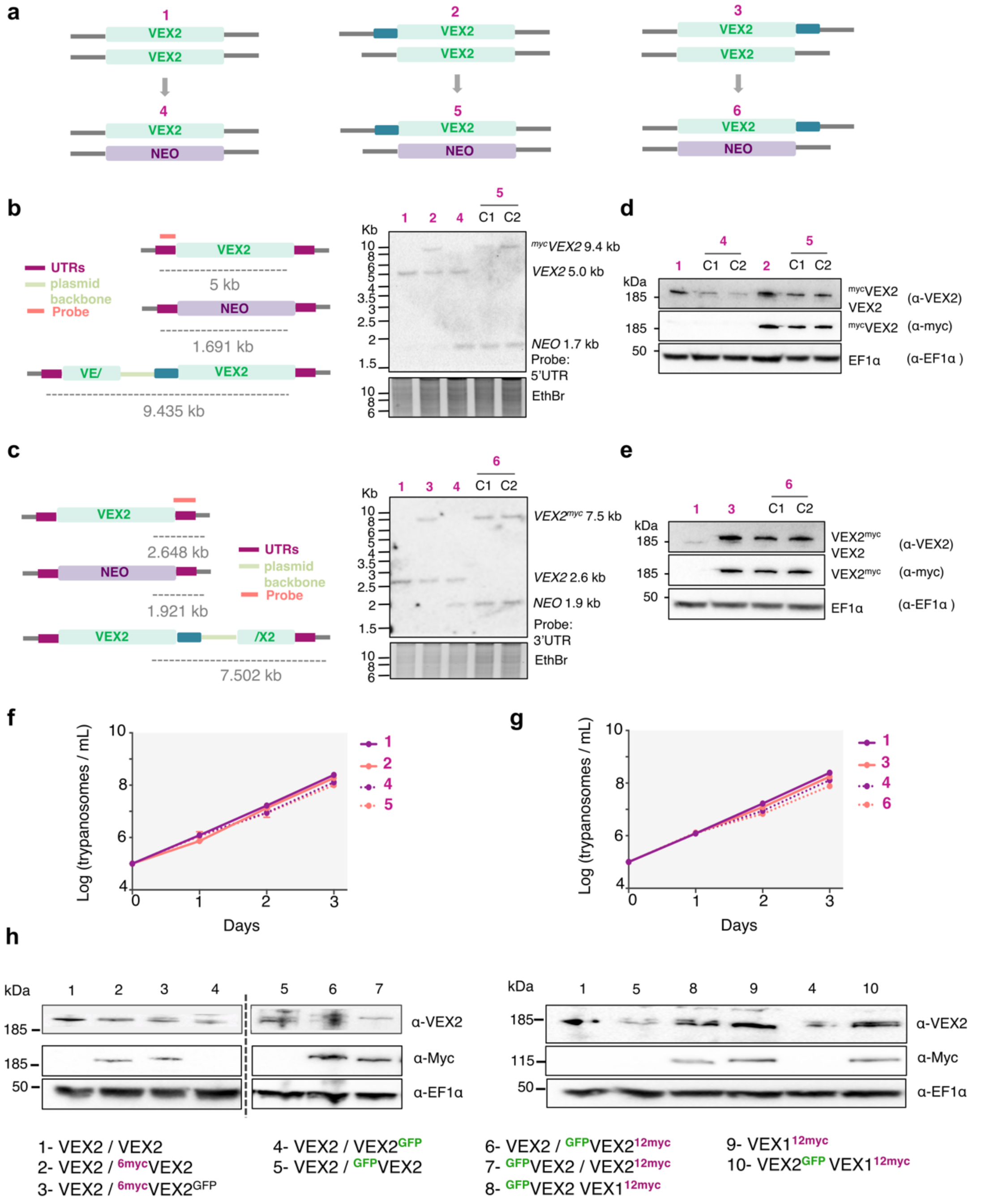

Supplementary Figure 1. **^{6myc}VEX2 and VEX2^{12myc} are functional.** In a cell line where one allele of VEX2 is tagged with myc at the N- or C-terminus, we knocked-out the second allele using a replacement cassette conferring resistance to neomycin (wild-type cells were transfected in parallel as a control). These transfectants were successfully generated as confirmed by Southern-blot and protein-blot, showing that VEX2 is functional. **a** Schematics illustrating the replacement of one *VEX2* allele by a replacement cassette containing *NEO* in wild-type cells (1) or cells where one VEX2 allele was endogenously tagged with 6xmyc at the N-terminus (2) or 12xmyc at the C-terminus (3). The resulting mutants were analysed by Southern-blot (**b-c**) and protein-blot (**d-e**). In **b** DNA was digested with AgeI and EcoRV,

whereas in **c**, DNA was digested with EcoRV and PsiI. In **d-e** both α-VEX2 (top panels) and α-myc (middle panels) antibodies were used. **f-g** Cumulative growth of the cell lines for which genotypes are illustrated in **a** In **b-g**, the genotypes illustrated in a are coloured in pink; in **b-e**, two biological replicates are depicted (C1 and C2). **h** Protein-blotting analysis of VEX1 and VEX2 tagged strains using a-VEX2 and a-myc. EF1α was used as a loading control in **d/e/h**.

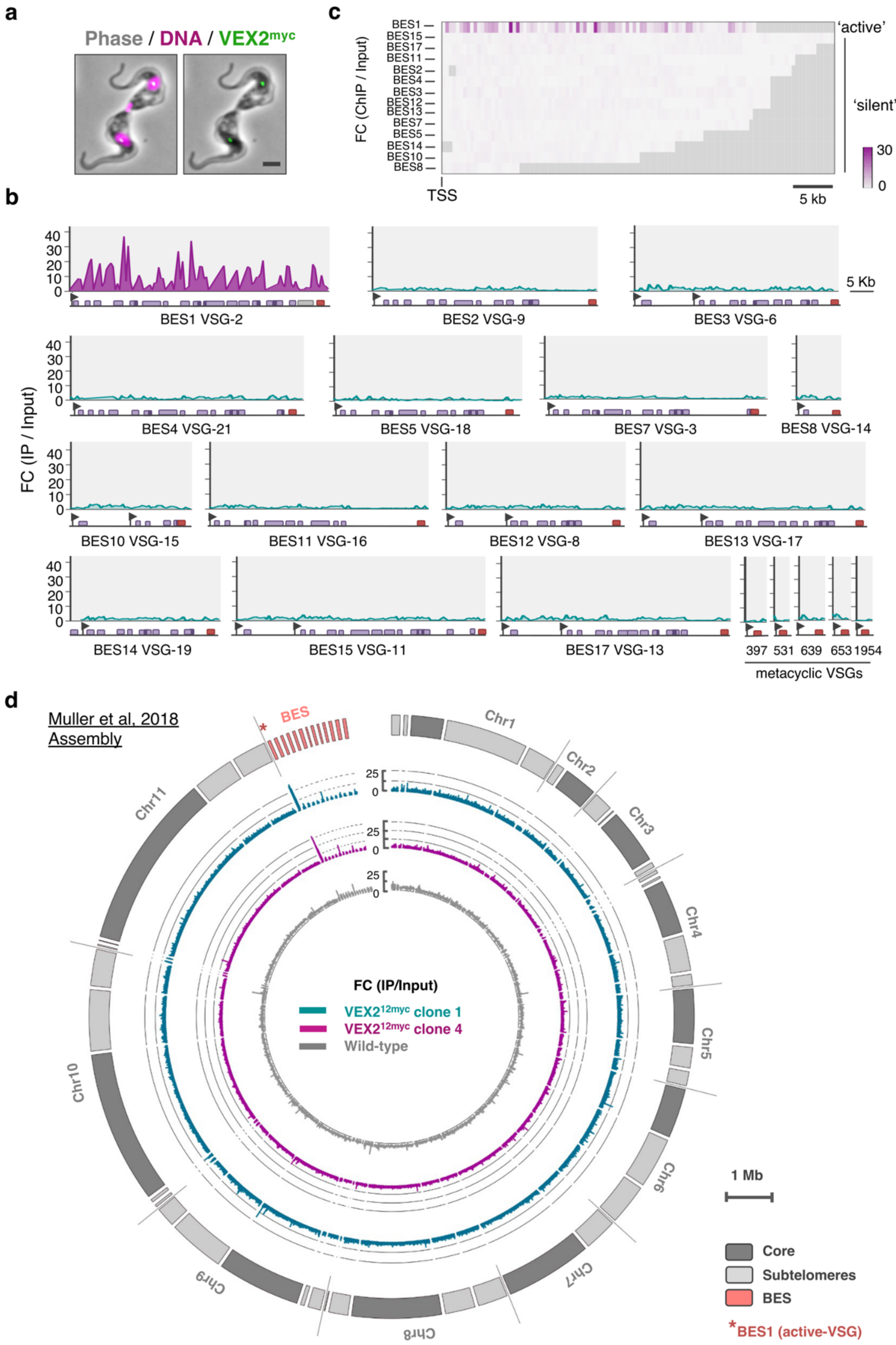

Supplementary Figure 2. **Supplementary ChIP-Seq dataset for VEX2 and *VSG*-ESs. a** Wide-field immunofluorescence microscopy analysis of VEX2^{12myc}. DNA was stained with DAPI, scale bar: 2 μm. **b** VEX2^{myc} chromatin-immunoprecipitation followed by next generation sequencing (ChIP-Seq). VEX2 enrichment across all *VSG*-ESs is shown as fold change (FC)

between ChIP *versus* input. Bin size 0.5 kb; values are averages of two biological replicates. PCR duplicates and reads that do not map uniquely were excluded from the analysis. **c** The heatmap depicts VEX2-enrichment across all *VSG*-ESs. TSS, transcription start site. The grey area indicates where shorter transcription units end. **d** The circos plot shows VEX2-enrichment (two biological replicates in purple and cyan) using a second genome sequence assembly, for *T. brucei* L427 2018, and for comparison with the reference genome assembly in Fig. 1a; the L427 assembly includes distinct hemizygous sub-telomeres from otherwise homologous chromosomes. The inner track shows data for a no myc-tag control (wild-type; grey); bin size 1 kb.

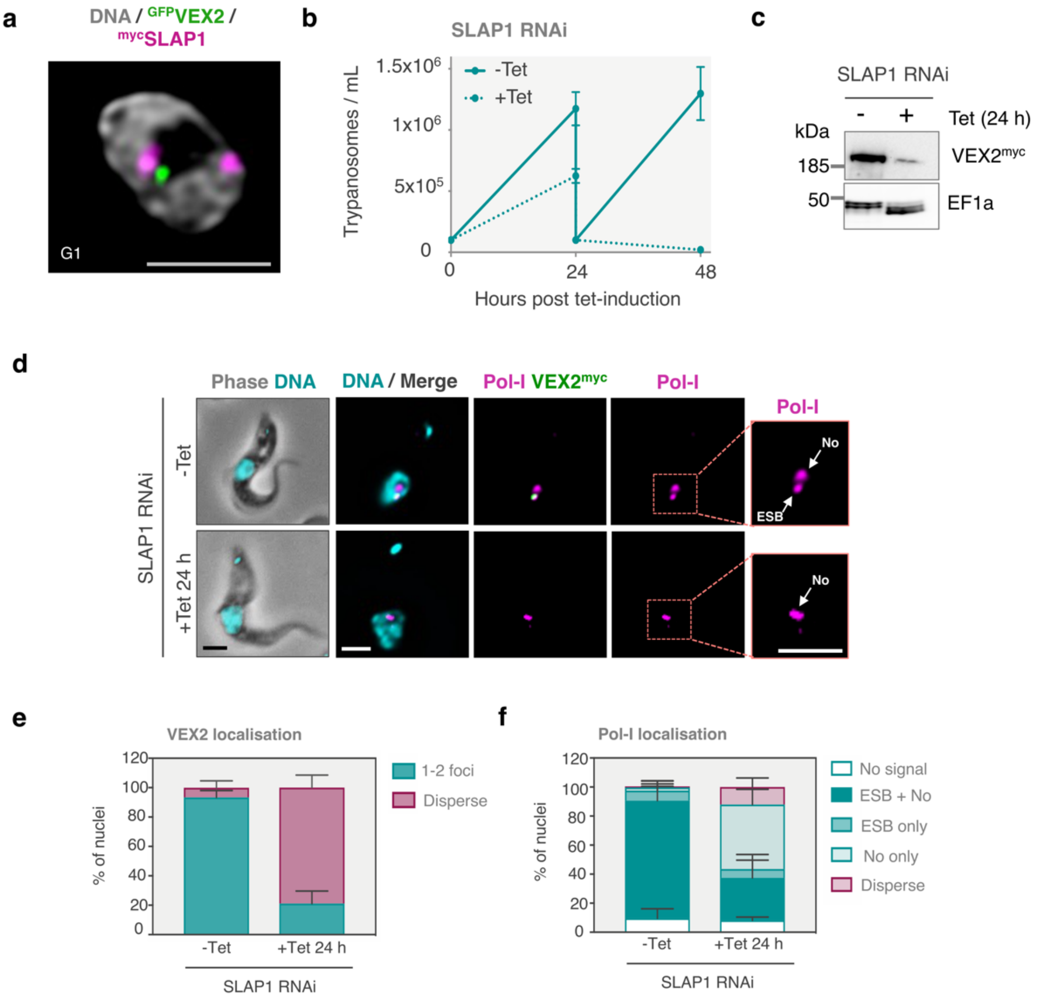

Supplementary Figure 3. **VEX2 sequestration requires SLAP1. a** Super-resolution microscopy analysis of ^{GFP}VEX2 / ^{myc}SLAP1. The images are representative, were acquired using a Zeiss Elyra 7 microscope (Lattice SIM 2) and correspond to 3D projections by brightest intensity of 0.1 μm stacks. **b-c** growth curve (**b**) and protein-blotting analysis of VEX2^{myc} expression (**c**) following SLAP1 knockdown by RNAi. **d-f** Wide-field immunofluorescence microscopy analysis of VEX2^{myc} (green) and Pol-I (magenta) localisation following SLAP1 knockdown. No, nucleolus; ESB, expression-site body. **e-f** The graphs depict averages of two (**e**) or three (**f**) biological replicates and representative of two independent experiments; >100 nuclei were considered per condition; error bars correspond to standard deviation. **a, d** DNA was stained with DAPI (grey, cyan); scale bars: 2 μm.

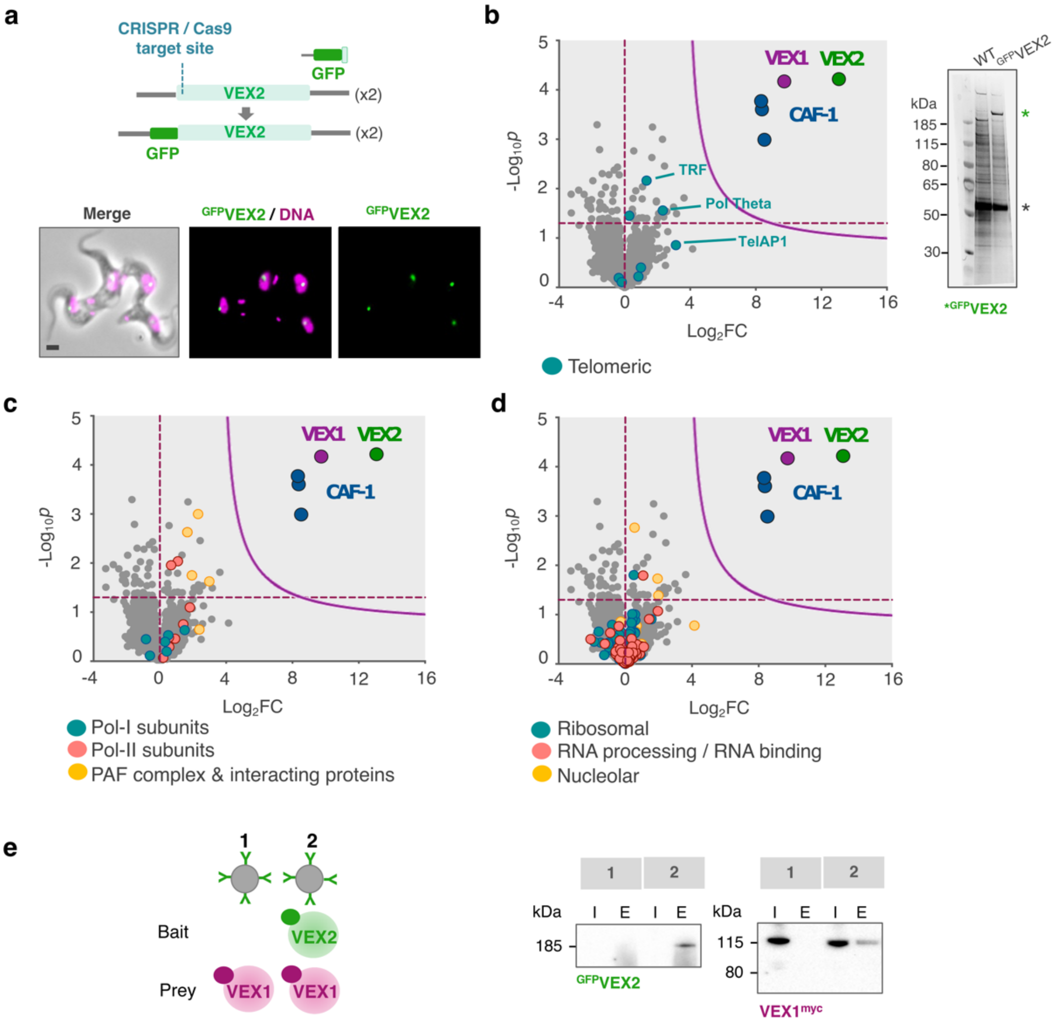

Supplementary Figure 4. **^{GFP}VEX2 affinity purification identified VEX1 and CAF-1 as the major interacting partners. a** VEX2 double allele tagging with GFP at the N-terminus was engineered using CRISPR/Cas9. Wide-field immunofluorescence microscopy analysis shows correct localisation of ^{GFP}VEX2. **b-d** ^{GFP}VEX2 affinity purification using a cryo-grinding approach followed by LC-MS/MS analysis. The volcano plots depict log2 fold change (log2FC) versus -log10*p* (statistical significance), comparing the tagged cell line with a wild-type control (untagged). *p* values were determined using a two-sided two sample t-test (Perseus v1.5.2.6). Cutoff curves were defined by a permutation-based false discovery rate of 5% and minimum fold change S_0 at 2.0. The data correspond to three independent replicates, using sodium chloride / tween-20 as extraction buffer. Different protein cohorts are highlighted in cyan, salmon or yellow. **e** Co-immunoprecipitation of ^{GFP}VEX2/VEX1^{myc} with α-GFP (Abcam) conjugated to magnetic Dynabeads followed by protein-blot analysis; lysis was in RIPA buffer. Green circle, GFP; magenta circles, myc; I, input; E, elution.

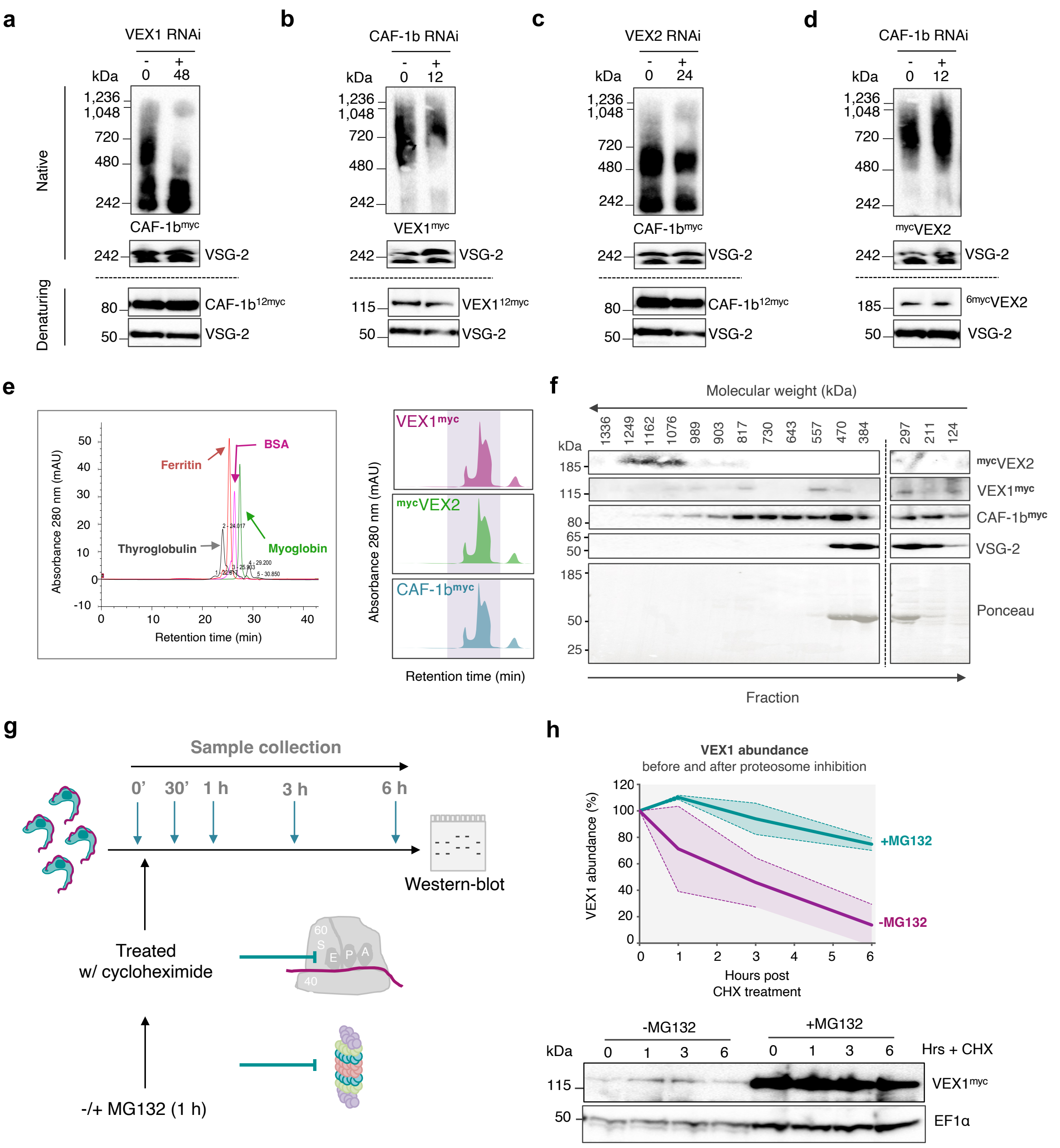

Supplementary Figure 5. **Analysis of the VEX-complex components. a-d** CAF-1b^{myc} migration following VEX1 knockdown (**a**), VEX1^{myc} migration following CAF-1b knockdown (**b**), CAF-1b^{myc} migration following VEX2 knockdown (**c**), ^{myc}VEX2 migration following CAF-1b knockdown (**d**). Both native-PAGE (upper panels) and SDS-PAGE (lower panels) anti-myc blots are shown. VSG-2 was used as control. The blots are representative of at least two independent experiments. **e-f** Size exclusion chromatography analysis of trypanosome extracts expressing VEX1^{myc}, ^{myc}VEX2 and CAF-1b^{myc} at endogenous loci. **e** The chromatogram of molecular standards that were used to generate a calibration curve correlating elution volume with MW. **f** chromatograms of the three different cell extracts are depicted on the left. The region with the purple shading corresponds to the interval where the fractions were collected. The collected fractions were analysed by protein-blot (right panels). Tracks for VEX1^{myc}, ^{myc}VEX2 and CAF-1b^{myc} correspond to independent gels. VSG-2 and

ponceau staining were used as controls. The data are representative of three independent experiments. Consistent with native gel analysis, the size exclusion data reveal VEX2 mostly detected in very large oligomeric forms up to 1.2 megadaltons. VEX1 can be detected as a monomer (124 kDa), a dimer (211-297 kDa), but mostly in two larger pools of 384-557 kDa and 817-1076 kDa. CAF-1 can be detected on its own (124-297 kDa), but also at higher MW (384-1076 kDa). **g-h** VEX1^{myc} turnover experiments**. g** The schematic shows the experimental procedure. VEX1^{myc} turnover was monitored following cycloheximide (CHX) treatment (100 μg/mL for 0 to 6 h at 37°C) and proteosome inhibition by MG132 (5 μM for 1 h, at 37°C; **h**). A representative protein-blot is shown. EF1α was used as a loading control. The graph shows an average of 2 independent experiments; the solid line shows the average, the dashed lines show the upper and lower limits of the standard deviation.

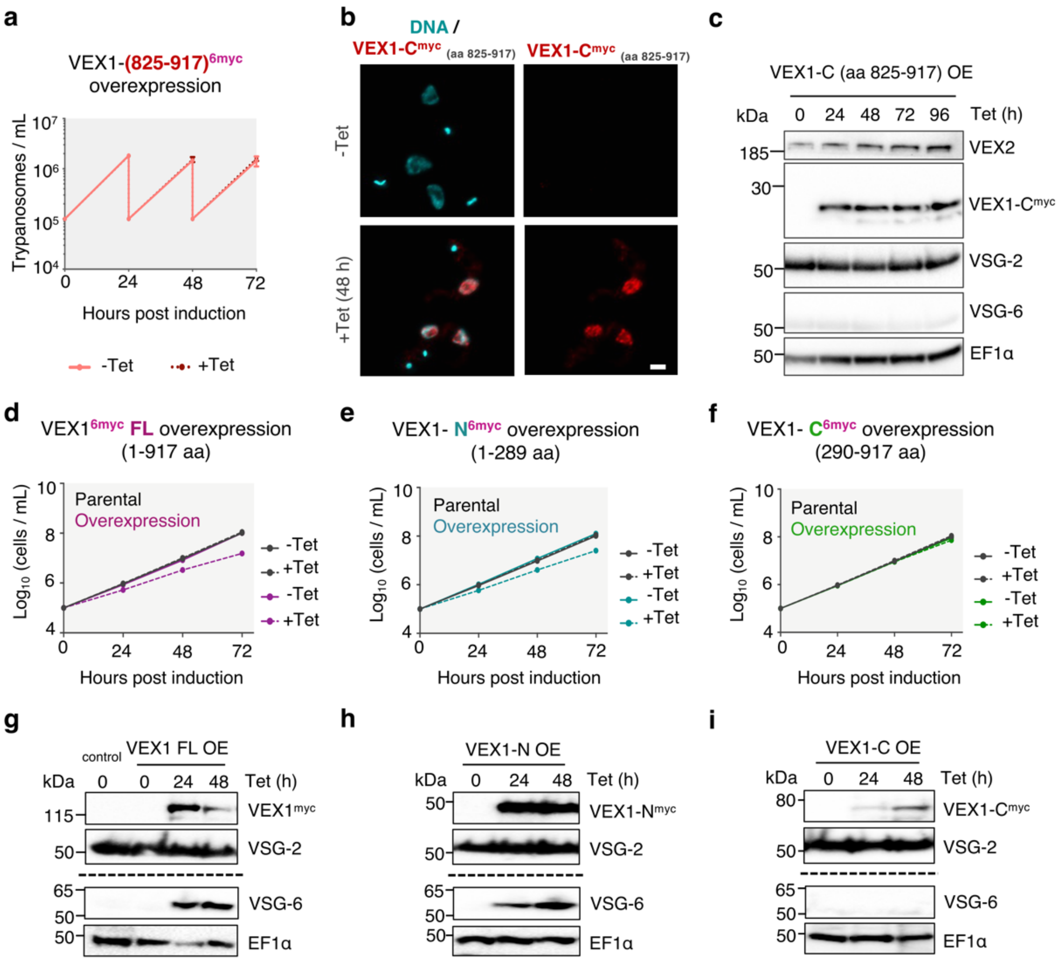

Supplementary Figure 6. **Phenotypes associated with overexpression of VEX1 fragments. a-c** Tet-inducible overexpression of VEX1 (aa 825-917) fused with 6xmyc. Cumulative growth of the cell lines before and after tetracycline induction (**a**). Protein-blotting analysis of VEX1 (aa 825-917) and VSG-2 and VSG-6 expression (**c**). **b** Wide-field immunofluorescence microscopy analysis to assess VEX1 (aa 825-917) expression following Tet-inducible overexpression (48 h). DNA was counter-stained with DAPI; Scale bar, 2 μ m. **d-i** Tet-inducible overexpression of full-length VEX1, N- or C-terminal regions fused with 6xmyc. **d-f** Cumulative growth of the cell lines before and after tetracycline induction. **g-i** Protein-blotting analysis of full-length VEX1, N- or C-terminal regions, and VSG-2 and VSG-6 expression. EF1α was used as a loading control in **c/g-i**. Data in **a/d-f** are averages of two biological replicates.

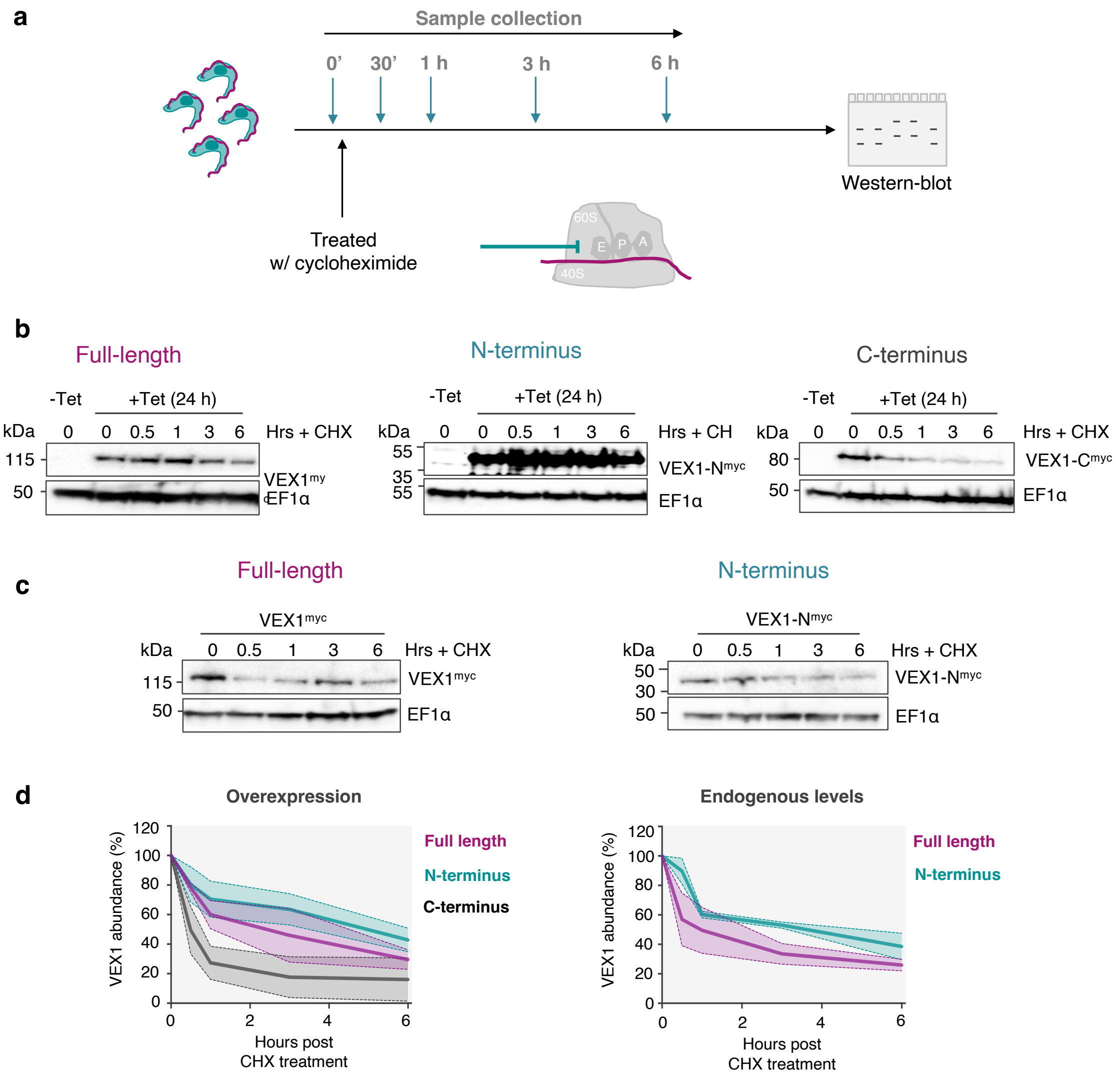

Supplementary Figure 7. **The C-terminal region of VEX1 controls protein turnover. a** A schematic of the experimental procedure. Full-length VEX1, and N-or C-terminal region (fused with 6xmyc) turnover was monitored following cycloheximide treatment (100 μg/mL for 0 to 6 h at 37°C) and tet-inducible overexpression (24 h post induction, **b** & **d** left panel) or at endogenous levels (**c** & **d** right panel). Representative protein-blots are depicted in **b-c**. EF1α was used as a loading control on the same blots used for VEX1 analysis. The graphs in **d** show averages of 3 independent experiments; the solid line shows the average, the dashed lines show the upper and lower limits of the standard deviation.

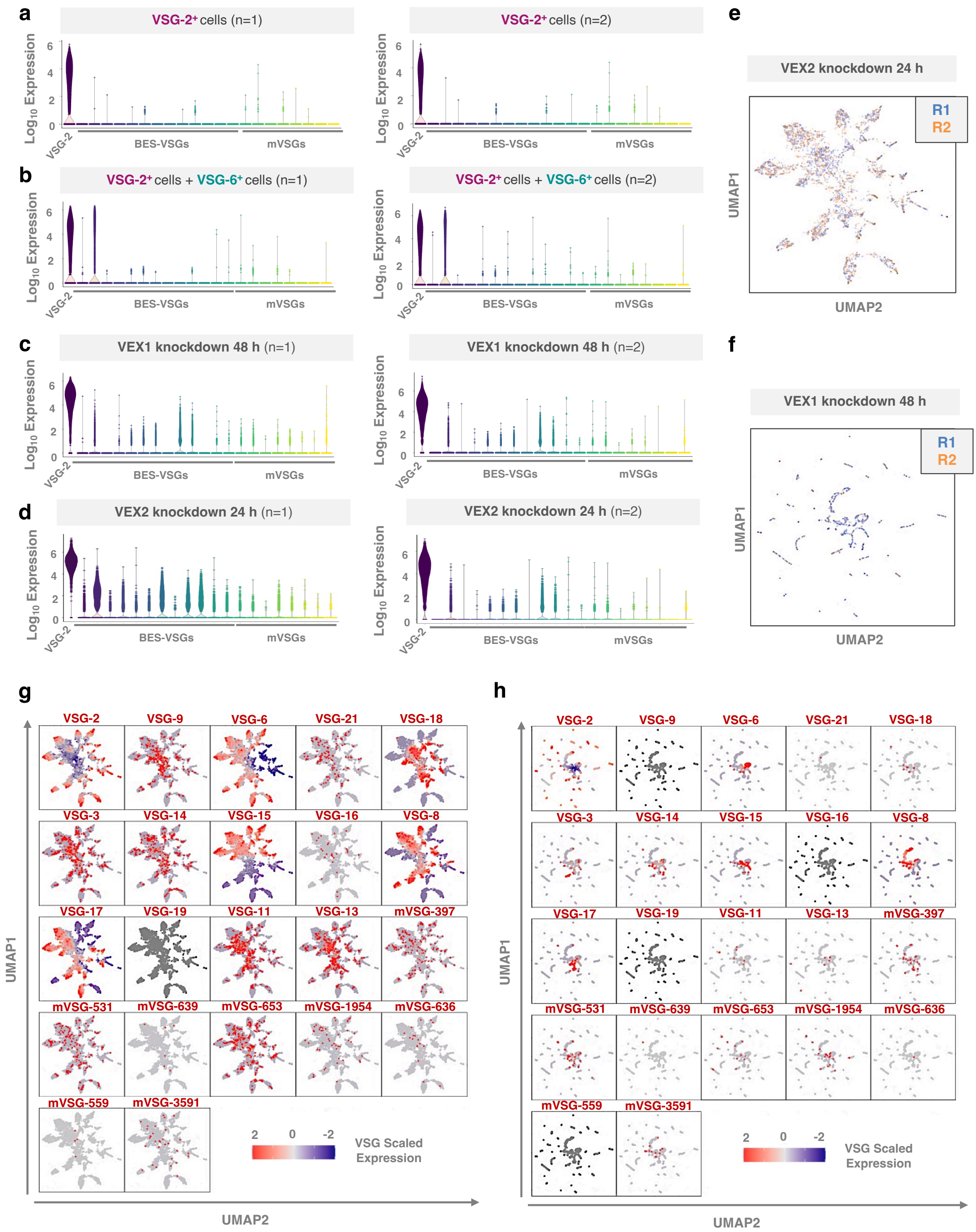

Supplementary Figure 8. **Supplementary *VSG* expression profiles following VEX1 or VEX2 knockdown and scRNA-Seq. a-d** Violin plots showing the expression of bloodstream and metacyclic *VSG* transcripts per cell in wild-type (**a**), a 1:1 mixture of VSG-2+ and VSG-6+ cells (**b**), VEX1-depleted cells (**c**) or VEX2-depleted cells (**d**). Two biological replicates are shown per condition. **e-f.** Low dimensional plot (UMAP) of each cell following VEX2 (**e**) or

VEX1 (**f**) knockdown (after filtering) in both biological replicates. Each point is the VSGome (sum of the *VSG* transcripts in the transcriptome) of one cell positioned according to similarity with neighbouring VSGomes, coloured by biological replicate. **g-h** UMAP of VEX2-depleted (**g**) or VEX1-depleted (**h**) parasite VSGomes coloured by transcript counts for VSG-2 ('active') and twenty-one additional VSGs, which are usually 'silent'. Expression levels are individually scaled for each *VSG* transcript, based on the corresponding minimum and maximum values; data corresponds to the combination of individual cells from both biological replicates.
