# Supplementary material for: An allele-selective inter-chromosomal protein bridge supports monogenic antigen expression in the African trypanosome
Source: Nat Commun. 2023 Dec 11;14:8200. doi: 10.1038/s41467-023-44043-y (PMC10713589; doi:10.1038/s41467-023-44043-y)
Supplement: Supplementary file 3 — Description of Additional Supplementary Files [file 41467_2023_44043_MOESM3_ESM.pdf]

Description of Additional Supplementary Files

File Name: Supplementary Data 1
Description: Excel file with 6 datasheets. Sheets 1-2: VEX2-12myc ChIP-Seq Sheet 3-4: GFP-VEX2 Pull-down Sheet 5: Primers & oligonucleotides Sheet 6: Resources Table
