# Supplementary material for: An allele-selective inter-chromosomal protein bridge supports monogenic antigen expression in the African trypanosome
Source: Nat Commun. 2023 Dec 11;14:8200. doi: 10.1038/s41467-023-44043-y (PMC10713589; doi:10.1038/s41467-023-44043-y)
Supplement: Supplementary file 5 — Reporting Summary [file 41467_2023_44043_MOESM5_ESM.pdf]

nature portfolio

Corresponding author(s): David Horn / Joana Faria

Last updated by author(s): 23rd November 2023

Reporting Summary

Nature Portfolio wishes to improve the reproducibility of the work that we publish. This form provides structure for consistency and transparency in reporting. For further information on Nature Portfolio policies, see our Editorial Policies and the Editorial Policy Checklist.

Statistics

For all statistical analyses, confirm that the following items are present in the figure legend, table legend, main text, or Methods section.

n/a	Confirmed	
☐	☒	The exact sample size (*n*) for each experimental group/condition, given as a discrete number and unit of measurement
☐	☒	A statement on whether measurements were taken from distinct samples or whether the same sample was measured repeatedly
☐	☒	The statistical test(s) used AND whether they are one- or two-sided<br>*Only common tests should be described solely by name; describe more complex techniques in the Methods section.*
☒	☐	A description of all covariates tested
☒	☐	A description of any assumptions or corrections, such as tests of normality and adjustment for multiple comparisons
☐	☒	A full description of the statistical parameters including central tendency (e.g. means) or other basic estimates (e.g. regression coefficient) AND variation (e.g. standard deviation) or associated estimates of uncertainty (e.g. confidence intervals)
☐	☒	For null hypothesis testing, the test statistic (e.g. *F*, *t*, *r*) with confidence intervals, effect sizes, degrees of freedom and *P* value noted<br>*Give P values as exact values whenever suitable.*
☒	☐	For Bayesian analysis, information on the choice of priors and Markov chain Monte Carlo settings
☒	☐	For hierarchical and complex designs, identification of the appropriate level for tests and full reporting of outcomes
☐	☒	Estimates of effect sizes (e.g. Cohen's *d*, Pearson's *r*), indicating how they were calculated

Our web collection on statistics for biologists contains articles on many of the points above.

Software and code

Policy information about availability of computer code

Data collection	Leica Application Suite X (LASX) / Leica / https://www.leica-microsystems.com/products/microscope-software/details/product/leica-las-x-ls/<br>Zen Pro / Zeiss / https://www.zeiss.com/microscopy/int/products/microscope-software/zen.html
Data analysis	Graphpad Prism Software v9.0 / Graphpad Software / https://www.graphpad.com/scientific-software/prism/<br>Bowtie2 / Langmed and Salzberg, 2012 / http://bowtie-bio.sourceforge.net/bowtie2/index.shtml<br>SAMtools / Li et al, 2009 / http://samtools.sourceforge.net/<br>Artemis / Rutherford et al, 2011 / https://www.sanger.ac.uk/science/tools/artemis<br>Deeptools2 / Ramirez et al, 2016 / https://deeptools.readthedocs.io/en/develop/<br>Bedtools / Quinlan and Hall, 2010 / http://bedtools.readthedocs.io/en/latest/<br>Picard Tools / Picard Tools / https://broadinstitute.github.io/picard/<br>Circos / Krzywinski et al, 2009 / http://circos.ca<br>Galaxy / Galaxy / https://usegalaxy.org<br>MaxQuant / MaxQuant / https://www.maxquant.org/<br>Perseus / Tyanova et al, 2016 / http://coxdocs.org/doku.php?id=perseus:start<br>BoxPlotR / Spitzer et al, 2014 / http://shiny.chemgrid.org/boxplotr/<br>Heatmapper / Babicki et al, 2016 / http://www.heatmapper.ca/<br>CellRanger / 10x Genomics / https://support.10xgenomics.com/single-cell-gene-expression/software/pipelines/latest/what-is-cell-ranger<br>LoupeBrowser / 10x Genomics / https://support.10xgenomics.com/single-cell-gene-expression/software/visualization/latest/what-is-loupe-cell-browser<br>Rstudio / Rstudio / https://www.rstudio.com<br>scRNA-Seq / (for Rstudio) / https://bioconductor.org/packages/release/data/experiment/html/scRNAseq.html

Seurat / Stuart et al, 2019 / https://satijalab.org/seurat/
SoupX / Young et al, 2020 / https://cran.r-project.org/web/packages/SoupX/index.html
Adobe Illustrator / Adobe Software / https://www.adobe.com/uk/products/illustrator.html
Fiji / Schindelin et al, 2012 / https://fiji.sc/

Custom code used in this study has been deposited in GitHub (https://github.com/mtinti/VSG_single_cell) and in Zenodo DOI: 10.5281/zenodo.10061206.

For manuscripts utilizing custom algorithms or software that are central to the research but not yet described in published literature, software must be made available to editors and reviewers. We strongly encourage code deposition in a community repository (e.g. GitHub). See the Nature Portfolio guidelines for submitting code & software for further information.

Data

Policy information about availability of data

All manuscripts must include a data availability statement. This statement should provide the following information, where applicable:

- Accession codes, unique identifiers, or web links for publicly available datasets
- A description of any restrictions on data availability
- For clinical datasets or third party data, please ensure that the statement adheres to our policy

scRNA-Seq and ChIP-Seq data available at NCBI BioProject (Project ID PRJNA942067). LC-MS/MS data available at ProteomeXchange (Accession No PXD040686). Trypanosoma brucei L427 reference proteome was downloaded from TriTrypDB (https://tritrypdb.org/tritrypdb/app).

Research involving human participants, their data, or biological material

Policy information about studies with human participants or human data. See also policy information about sex, gender (identity/presentation), and sexual orientation and race, ethnicity and racism.

Reporting on sex and gender	N/A
Reporting on race, ethnicity, or other socially relevant groupings	N/A
Population characteristics	N/A
Recruitment	N/A
Ethics oversight	N/A

Note that full information on the approval of the study protocol must also be provided in the manuscript.

Field-specific reporting

Please select the one below that is the best fit for your research. If you are not sure, read the appropriate sections before making your selection.

☒ Life sciences ☐ Behavioural & social sciences ☐ Ecological, evolutionary & environmental sciences

For a reference copy of the document with all sections, see nature.com/documents/nr-reporting-summary-flat.pdf

Life sciences study design

All studies must disclose on these points even when the disclosure is negative.

Sample size	Sample size was not statistically predetermined for the individual experiments. The sample size is appropriate as we were able to robustly detect differences as low as 10% (versus control) for different biological replicates and independent experiments.<br>To reduce the influence of noise on the statistical analysis of omics data, technical replicate measurements are a common approach, e.g. for mass spectrometry data collecting three technical replicates per biological sample is typical. For ChIP-Seq and scRNA-Seq experiments, two biological replicates were performed. For microscopy analyses, where possible, we typically quantified >100 cells.
Data exclusions	In Fig. 2, Fig. 4b-e, Fig. 5a and Supp Fig. 3e, cells displaying no detectable signal by visual examination were excluded, but never more than 10%.
Replication	All attempts of replication were successful. scRNA-Seq and ChIP-Seq experiments were performed in biological duplicates for each cell line. Proteomics experiments were performed in triplicates. For super resolution microscopy analyses, typically 100 cells were analysed per replicate for total cells or specific cell cycle phases, respectively. For each immunofluorescence experiment, 2 or more independent experiment with 2 or 3 biological replicates were performed. For all the biochemical analyses (native gels, gel filtration, protein turnover determination, Co-IPs), two or three independent experiments were conducted.
Randomization	Two or more independent biological replicates were typically analyzed and behaved similarly, which was considered sufficient for cross-

Randomization	validation - randomization was therefore not considered necessary.
Blinding	Blinding was not considered necessary since the scRNA-seq, ChIP-seq and proteomics analyses were automated. For the microscopy analyses, blinding was not considered necessary since they were conducted with multiple biological replicates and colocalisation assessed by Pearson's correlation coefficient. Overlapping, adjacent and separate foci presented a Pearson's correlation coefficient in the ranges ≥0.5 to ≤1, ≥−0.5 to <0.5 and ≥−1 to <−0.5, respectively.

Reporting for specific materials, systems and methods

We require information from authors about some types of materials, experimental systems and methods used in many studies. Here, indicate whether each material, system or method listed is relevant to your study. If you are not sure if a list item applies to your research, read the appropriate section before selecting a response.

Materials & experimental systems

n/a	Involved in the study
☐	☒ Antibodies
☐	☒ Eukaryotic cell lines
☒	☐ Palaeontology and archaeology
☒	☐ Animals and other organisms
☒	☐ Clinical data
☒	☐ Dual use research of concern
☒	☐ Plants

Methods

n/a	Involved in the study
☐	☒ ChIP-seq
☒	☐ Flow cytometry
☒	☐ MRI-based neuroimaging

Antibodies

Antibodies used

ANTIBODY / SOURCE / IDENTIFIER
Mouse anti-Myc 9B11 (1:2,000 for immunofluorescence, 10 µg per Co-IP) / New England Biolabs / Cat# 2276 RRID:AB_331783
Rabbit anti-Myc 71D10 (1:500 for IFA) / New England Biolabs / Cat# 2278 RRID:AB_490778
Mouse anti-Myc 4A6 (1:10,000 for Western-blotting) / Merck-Millipore / Cat# 05-724 RRID:AB_568800
Rabbit anti-GFP (1:250 for immunofluorescence) / ThermoFisher Scientific / Cat# A-6455 RRID:AB_221570
Rabbit anti-GFP (1:500 for immunofluorescence; 1 µg per Co-IP; 1:1,000 for WB) / Abcam / Cat# Ab290 RRID:AB_303395
Rabbit anti-Pol-I (IFA + WB), validation available in Glover et al, 2016 (doi: 10.1073/pnas.1600344113)
GFP-Trap-MA / Chromotek / Cat# gtma-10
Mouse anti-EF1α CBP-KK1 (1:20,000 for Western-blotting) / Merck-Millipore / Cat# 05-235 RRID:AB_309663
Rat anti-VSG-2 (1:10,000 for immunofluorescence) / Prof. George Cross, Rockefeller University, Hoek and Cross, 1999/ N/A
Rabbit anti-VSG-2 (1:20,000 for Western-blotting) / Prof. George Cross, Rockefeller University, Hoek and Cross, 1999 / N/A
Rabbit anti-VSG-6 (1:10,000 for immunofluorescence) / Prof. George Cross, Rockefeller University, Hoek and Cross, 1999 / N/A
Rabbit anti-Histone H3 (1:5,000 for Western-blotting) / Abcam / Cat#ab1791 RRID:AB_302613
Mouse monoclonal anti-digoxigenin 21H8 (1:10,000 for DNA-FISH) / Abcam / Cat# Ab420 RRID:AB_304362
Streptavidin, Alexa Fluor 488 conjugate / ThermoFisher Scientific / Cat# S32354 RRID:AB_2315383
Rabbit anti-VEX2 (1:1,000 for Western-blotting) / Custom made by Thermo Scientific (Faria et al, 2019) / N/A
Goat anti-mouse Alexa 488 (1:1,000 or 1:2,000 for immunofluorescence) / ThermoFisher Scientific / Cat# A-11001 RRID:AB_2534069
Goat anti-rabbit Alexa 488 (1:1,000 or 1:2,000 for immunofluorescence; 1:2,000 for FACS) / ThermoFisher Scientific / Cat# A-11034 RRID:AB_2576217
Goat anti-mouse Alexa 568 (1:1,000 or 1:2,000 for immunofluorescence) / ThermoFisher Scientific / Cat# A-11004 RRID:AB_2534072
Goat anti-rabbit Alexa 568 (1:1,000 or 1:2,000 for immunofluorescence) / ThermoFisher Scientific / Cat# A-11011 RRID:AB_143157
Goat anti-rabbit Alexa 647 (1:1,000 for immunofluorescence) / ThermoFisher Scientific / Cat# A-21244 RRID:AB_2535812
Chicken anti-rat Alexa 488 (1:1,000 for immunofluorescence) / ThermoFisher Scientific / Cat# A-21470 RRID:AB_2535873
Goat anti-rat Alexa 647 (1:2,000 for FACS) / ThermoFisher Scientific / Cat# A-21247 RRID:AB_141778
Goat anti-mouse HRP (1:2,000) / Biorad / Cat# 1721011 RRID:AB_11125936
Goat anti-rabbit HRP (1:2,000) / Biorad / Cat# 1706515 RRID:AB_11125142

Validation

There were no new antibody specifically generated for this study. All antibodies have been widely used and validated for the purposed reported here.

Eukaryotic cell lines

Policy information about cell lines and Sex and Gender in Research

Cell line source(s)	Trypanosoma brucei brucei Lister 427 was originally obtained from Prof. George Cross (Rockefeller University, NYC, USA). Subsequent genetic modifications were performed by the authors - see complete list of cell lines in Supp Data Sheet 6.
Authentication	scRNA-seq, ChIP-seq, proteomics and microscopy provided authentication.
Mycoplasma contamination	Mycoplasma contamination check carried our approx. every 3 years - no positive results from those tests to date.
Commonly misidentified lines (See ICLAC register)	T. b. brucei L427 is not a commonly misidentified line.

Plants

Seed stocks	N/A
Novel plant genotypes	N/A
Authentication	N/A

ChIP-seq

Data deposition

☒ Confirm that both raw and final processed data have been deposited in a public database such as GEO.

☐ Confirm that you have deposited or provided access to graph files (e.g. BED files) for the called peaks.

Data access links<br>*May remain private before publication.*	ChIP-Seq data available at NCBI BioProject (Project ID PRJNA942067).
Files in database submission	Fastq files wew deposited as follows:<br>T. brucei L427 VEX2-12myc clone 1 (Input & ChIP)<br>T. brucei L427 VEX2-12myc clone 6 (Input & ChIP)<br>T. brucei L427 wild-type untagged (Input & ChIP)<br>Enrichment traces for VSG expression sites and core genome were provided in Supplementary File Sheet 3.
Genome browser session<br>(e.g. UCSC)	Not applicable

Methodology

Replicates	Two biological replicates.
Sequencing depth	Approx. 20 million reads per sample.
Antibodies	α-Myc antibody (NEB, clone 9B11) coupled to Dynabeads Protein G (2.8μm).
Peak calling parameters	Peak calling was conducted using MACS2 – a threshold (minimum p and q values to define a peak) was defined by comparison with the untagged control.
Data quality	Reads per CDS feature were extracted using featureCount, normalised to the total amount of reads per sample, and then used to calculate the ratio of ChIP/Input.
Software	Bowtie 2-mapping was with the parameters --very-sensitive --no-discordant --phred33. Alignment files were manipulated with SAMtools. Alignments were inspected with the Artemis genome browser. The resulting BAM files were loaded into Galaxy for downstream analysis. PCR duplicate reads were removed using Picard MarkDuplicates (https://broadinstitute.github.io/picard/) and the reads were filtered with mapQ > 0 and mapQ > 1 for core genome and VSG-ESs, respectively. The ratio of ChIP/Input across the genome was generated using BamCompare (deepTools) with a bin size of 500 bp or 1 kb, generating bedgraph files as outputs.
